# Antagonistic lactic acid bacteria isolated from goat milk and identification of a novel nisin variant *Lactococcus lactis*

**DOI:** 10.1186/1471-2180-14-36

**Published:** 2014-02-12

**Authors:** Luana Martins Perin, Luís Augusto Nero

**Affiliations:** 1 Departamento de Veterinária, Universidade Federal de Viçosa, Viçosa, Minas Gerais 36570 000, Brazil

## Abstract

**Background:**

The raw goat milk microbiota is considered a good source of novel bacteriocinogenic lactic acid bacteria (LAB) strains that can be exploited as an alternative for use as biopreservatives in foods. The constant demand for such alternative tools justifies studies that investigate the antimicrobial potential of such strains.

**Results:**

The obtained data identified a predominance of *Lactococcus* and *Enterococcus* strains in raw goat milk microbiota with antimicrobial activity against *Listeria monocytogenes* ATCC 7644. Enzymatic assays confirmed the bacteriocinogenic nature of the antimicrobial substances produced by the isolated strains, and PCR reactions detected a variety of bacteriocin-related genes in their genomes. Rep-PCR identified broad genetic variability among the *Enterococcus* isolates, and close relations between the *Lactococcus* strains. The sequencing of PCR products from *nis*-positive *Lactococcus* allowed the identification of a predicted nisin variant not previously described and possessing a wide inhibitory spectrum.

**Conclusions:**

Raw goat milk was confirmed as a good source of novel bacteriocinogenic LAB strains, having identified *Lactococcus* isolates possessing variations in their genomes that suggest the production of a nisin variant not yet described and with potential for use as biopreservatives in food due to its broad spectrum of action.

## Background

Goat milk is the second variety of milk most produced in the world [1]. Their production is increasing mainly because it could be an alternative to substitute the consumption of cow milk, due to evidences that it does not induce allergies, presents high digestibility, and also possess high nutritional quality [2]. As cow milk, goat milk has a very rich and complex autochthonous microbiota, and its detailed knowledge is essential for a future use of this matrix for the production of fermented products [3,4]. The main responsible for the natural fermentation of these products are microorganisms from the Lactic Acid Bacteria (LAB) group, that are widely studied due to their potential use as adjuvants and biopreservatives in foods [3,5-8]. Many studies already demonstrated that BAL has considerable inhibitory activity against pathogenic and spoilage microorganisms in foods [7-12], mainly by the production of bacteriocins [13,14].

Bacteriocins are small peptides that present antimicrobial activity and are of particular interest to food industries, representing natural alternatives to improve the safety and quality of foods [13,15]. Considering these characteristics, new bacteriocinogenic LAB strains and their bacteriocins are continuously searched, however only nisin and pediocin are the bacteriocins allowed to be applied in food, including cheeses [15,16]. Nisin is a lantibiotic produced by some *Lactococcus lactis* strains and up to now five nisin variants are already known: nisin A (the first to be discovered), Z, Q, U and F [17-19]. The differences between these variants are based on the changes in the amino acid chain, what could interfere in their antimicrobial activity.

The main sources of novel LAB strains capable of producing bacteriocins are food systems, mainly ones that are naturally contaminated with a diversity of microorganisms, such as animal origin products [9,20,21]. The production characteristics of meat and dairy products facilitate contamination by distinct microbial groups, determining a rich autochthonous microbiota in such food products. In this context, the autochthonous microbiota of raw goat milk is particularly interesting due to its diversity and the presence of several bacteriocinogenic LAB strains, as observed in previous studies [4,5,22,23]. Once isolated from a food sample, the antimicrobial activity of bacterocinogenic LAB strains must be properly characterized [24]. Relevant information that must be investigated includes possible bacteriocins the strains are able to produce, which can be assessed by the identification of specific genes related to known bacteriocins, followed by sequencing to identify variants [25,26]. Additionally, it is important to verify the inhibitory spectrum of the bacteriocins produced by newly isolated LAB strains. Such data can justify further studies with purified bacteriocins, in order to check a diversity of characteristics that allow their use in the food industry as biopreservatives.

The present study aimed to characterize the diversity of the main LAB groups that compose the autochthonous microbiota of raw goat milk and their bacteriocinogenic potential, in order to identify novel strains capable of producing known bacteriocin variants with potential application as biopreservatives.

## Methods

### Samples and microbiological analysis

Raw goat milk samples were collected from 11 goat farms (two samples per farm) located in Viçosa, Minas Gerais state, Brazil, and subjected to ten-fold dilution using 0.85% NaCl (w/v). Selected dilutions were pour plated in duplicate and in distinct culture media: M17 (Oxoid Ltd., Basingstoke, England, incubated at 35°C for 48 h, and at 42°C for 48 h), de Man, Rogosa and Sharpe (MRS) (Oxoid, incubated at 30°C for 48 h, under anaerobic conditions using GasPak EZ™ Gas Generating Container Systems, BD - Becton, Dickinson and Co., Franklin Lakes, NJ, USA), MRS at pH 5.5 (Oxoid, incubated at 35°C for 48 h, under anaerobic conditions using GasPak, BD), and Kanamycin Aesculin Azide (Oxoid, incubated at 35°C for 48 h). After incubation, colonies were enumerated and the results expressed as log colony-forming units per mL (log cfu/mL). From each culture media and sample, representative colonies were selected (about 10% of the observed count) and subjected to Gram staining and checked for catalase production. LAB characteristic colonies were subjected to addition microbiological analysis as described in the following sections.

### Antimicrobial activity and bacteriocin production

Isolates identified as LAB (Gram positive and catalase negative) were subjected to the spot-on-the-lawn method to identify their antimicrobial activity against *Listeria monocytogenes* ATCC 7644, according to CB Lewus, A Kaiser and TJ Montville [27]. Briefly, LAB isolates were cultured in MRS broth (Oxoid) at 35°C for 24 h, after which 1 μL aliquots were spotted on the surface of MRS agar (Oxoid) and incubated at 25°C for 24 h under anaerobic conditions (GasPak, BD); then, brain heart infusion (BHI, Oxoid) broth was added to bacteriological agar at 0.8% (w/v) and *L. monocytogenes* ATCC 7644 at 10^5^ cfu/mL was overlaid and incubated at 35°C for 24 h. The presence of inhibition halos was recorded as the antimicrobial activity of the tested isolate.

Isolates that presented antimicrobial activity were subjected to the spot-on-the-lawn protocol [27,28] to identify the bacteriocinogenic nature of their antimicrobial substances. For this, after the first incubation of the tested isolates in MRS plates, 2 mm diameter wells were cut adjacent to the colonies and enzyme solutions at 20 mg/mL were added: α-chimotrypsin, proteinase K, TPCK trypsin, α-amylase, papain, *Streptomyces griseus* protease, *Aspergillus niger* lipase, and lysozyme (all from Sigma-Aldrich, Inc., St. Louis, MO, USA). Half-moon halos associated with proteases were indicative of the bacteriocinogenic nature of the antimicrobial substances produced by the tested isolates.

### Molecular identification and rep-PCR fingerprinting of bacteriocinogenic isolates

Bacteriocinogenic isolates were cultured in MRS broth (Oxoid) at 35°C for 12 h, and the obtained cultures were subjected to DNA extraction using the Genomic Wizard DNA Purification Kit (Promega Corp., Madison, WI, USA).

Identification of these isolates was done by sequencing their 16S rRNA genes using the primers P1V1 and P4V3 (Table 1). Isolates identified as *Enterococcus* spp. were identified at the species level by sequencing the *pheS* (phenylalanyl-tRNA synthase α-subunit) gene using the primers pheS-21 and pheS-22 (Table 1). PCR reactions consisted of 25 μL of Go Taq Green Master Mix 2x (Promega), 10 pMol of each pair of primers, 2 μL of DNA (50 ng/μL) and ultra pure PCR water (Promega) to a final volume of 50 μL. PCR conditions for 16S rRNA were as described by N Klijn, AH Weerkamp and WM de Vos [29], and for *pheS* as detailed by SM Naser, FL Thompson, B Hoste, D Gevers, P Dawyndt, M Vancanneyt and J Swings [30], using the annealing temperatures described in Table 1. The PCR products (Table 1) were double-strand sequenced by Macrogen Inc. (Seoul, Korea), and the identification was given only for sequences with 100% of similarity when compared to the database of the National Center for Biotechnology Information (NCBI, http://www.ncbi.nlm.nih.gov/genbank) using the software Basic Alignment Search Tool (BLAST, http://p://www.ncbi.nlm.nih.gov/blast.cgi).

**Table 1:** Primers sequences, annealing temperatures, and expected fragment sizes of PCR reactions targeting specific genes for identification and bacteriocins encoding genes

**Target**	**Primers sequences (5'-sequence-3')**	**Fragment size (bp)**	**Annealing**	**Reference**
16S rRNA	GCGGCGTGCCTAATACATGC	700	42°C	[29]
	ATCTACGCATTTCACCGCTAC			
*phe*S	CAYCCNGCHCGYGAYATGC	470	46°C	[30]
	CCWARVCCRAARGCAAARCC			
*lan*B	TATGATCGAGAARYAKAWAGATATGG	400-500	40°C	[17,19]
	TTATTAIRCAIATGIAYDAWACT			
*lan*C	TAATTTAGGATWISYIMAYGG	200-300	40°C	[17,19]
	ACCWGKIIIICCRTRRCACCA			
*lan*M	ATGCWAGWYWTGCWCATGG	200-300	40°C	[17,19]
	CCTAATGAACCRTRRYAYCA			
*nis*	GGATAGTATCCATGTCTG	250	55°C	[31]
	CAATGATTTCGTTCGAAG			
*ent*A	CATCATCCATAACTATATTTG	126	56°C	[32]
	AAATATTATGGAAATGGAGTGTAT			
*ent*B	GAAAATGATCACAGAATGCCTA	162	58°C	[32]
	GTTGCATTTAGAGTATACATTTG			
*ent*P	TATGGTAATGGTGTTTATTGTAAT	120	58°C	[32]
	ATGTCCCATACCTGCCAAAC			
*ent*L50AB	STGGGAGCAATCGCAAAATTAG	98	56°C	[32]
	ATTGCCCATCCTTCTCCAAT			
*ent*AS48	GAGGAGTITCATGATTTAAAGA	340	56°C	[32]
	CATATTGTTAAATTACCAAGCAA			

Rep-PCR was performed according the protocol described by B Dal Bello, K Rantsiou, A Bellio, G Zeppa, R Ambrosoli, T Civera and L Cocolin [9] using a single primer (GTG)_5_ (5’-GTGGTGGTGGTGGTG-3’). PCR reactions contained 12.5 μL of Go Taq Green Master Mix 2x (Promega), 50 pMol of the primer, 2 μL of DNA (50 ng/μL) and ultra pure PCR water (Promega) to a final volume of 25 μL. PCR conditions were: 1) 5 min at 95°C, (2) 30 cycles of 30 s at 95°C; 30 s at 40°C and 8 min at 65°C, and (3) final extension of 16 min at 65°C. PCR products were electrophoresed in 2% (w/v) agarose gels for 6 h at a constant voltage of 75 V, in 0.5 × Tris/Borate/EDTA buffer (TBE). Gels were stained using GelRed (Biotium Inc., Hayward, CA, USA), and recorded using a transilluminator LPIX (Loccus Biotecnologia, São Paulo, SP, Brazil). Fingerprints were analysed using BioNumerics 4.6 (Applied Maths, Kortrijk, Belgium): The similarities among profiles were calculated using the Pearson correlation. Dendograms were constructed using the Unweighted Pair Group Method with Arithmetic Mean (UPGMA).

### Bacteriocin encoding genes

Bacteriocinogenic isolates were subjected to PCR to detect genes related to the expression of lantibiotics (*lan*B, *lan*C, and *lan*M), nisin (*nis*), and enterocins (A, P, B, L50A, L50B, and AS-48) using the primers presented in Table 1. PCR reactions consisted of 12.5 μL of Go Taq Green Master Mix 2x (Promega), 100 pMol of lantibiotics primers, or 60 pMol of nisin primers, or 10 pMol of enterocins primers, 1 μL of DNA (200 ng/μL), and ultra pure PCR water (Promega) to a final volume of 25 μL. All PCR reactions were conducted according the following conditions: 1) 95°C for 5 min, 2) 30 cycles at 95°C for 1 min, annealing temperature (Table 1) for 1 min, and 72°C for 1 min, and 3) final extension at 72°C for 10 min. The PCR products were electrophoresed in 1% (w/v) agarose gels in 0.5 × TBE, and stained in a GelRed bath (Biotium). Fragments with the specific expected sizes (Table 1) were recorded as positive results for each bacteriocin-encoding gene for each isolate. Positive results were confirmed by repeating the PCR reactions.

### Nisin gene sequencing and inhibitory spectrum of nisin positive isolates

PCR products of *nis*-positive isolates were sequenced by Macrogen Inc. The obtained results were analysed using the software Sequencher™ 4.1.4 (Technology Drive, Ann Arbor, MI, USA) in order to identify similarities between the translated amino-acid sequences and a nisin A, Z, Q, F or U sequences previously deposited in GenBank.

In addition, nisin-positive isolates were subjected to the spot-on-the-lawn protocol, as described previously [27], to identify their inhibitory activity against 22 target strains: 4 LAB, 4 *Listeria* spp., 2 *Pseudomonas* spp., 4 *Salmonella* spp., 6 *Staphylococcus* spp. and 2 *E. coli*. The diameters of the inhibition halos were measured to characterize the antimicrobial activities of the tested isolates.

## Results and discussion

### LAB counts of raw goat milk

The obtained mean counts for LAB of goat milk samples obtained by distinct culture media considered in the present study are presented in Table 2. The mean counts ranged from 3.07 to 3.89 log cfu/mL, and a total of 682 colonies was selected from the plated culture media, among which 423 were characterized as possessing typical LAB characteristics (Table 2). The majority of isolates from the LAB collection was characterized as cocci (377), a group described as the predominant component of raw milk microbiota [21,33]. The obtained results also highlighted the absence of adequate selectivity in the employed culture media, even for LAB (Table 2), necessitating further phenotypic analysis for proper characterization of the isolates [34]. The autochthonous microbiota of the goat milk could have originated mainly from utensils and environmental conditions, being highly influenced by the hygienic procedures of milking [35-37]. The method of storage also has a direct impact on the microbiota of raw milk, high temperatures being determinant for the predominance of lactococci [33].

**Table 2:** Mean counts and numbers of obtained isolates from distinct culture media used to enumerate presumptive lactic acid bacteria (LAB) groups from raw goat milk samples, and their typical LAB characteristics, antimicrobial activity, and sensitivity to eight distinct enzymatic solutions

**Results**	**Group**	**Culture media (incubation condition)**^**a**^					**Total**
		**M17 (35°C)**	**MRS (pH 5.5)**	**KAA**	**M17 (42°C)**	**MRS**	
Mean count (log cfu/mL)		3.89	3.47	3.07	3.65	3.61	-
Obtained isolates (n)	--	134	138	142	128	140	682
Typical LAB	Gram positive cocci, catalase negative	57	79	108	46	87	377
	Gram positive bacilli, catalase negative	7	18	4	5	12	46
Antimicrobial activity^b^	--	13	4	23	7	10	57
Enzymatic sensitivity^b^	α-chimotrypsin	9	2	13	7	6	37
	Proteinase K	11	1	18	6	10	46
	TPCK trypsin	10	3	10	5	10	38
	α-amylase	3	0	1	0	3	7
	Papain	4	3	8	3	6	24
	*Streptomyces griseus* protease	13	4	18	4	10	49
	*Aspergillus niger* lipase	9	2	6	4	7	28
	lysozyme	1	0	1	0	0	2

^a^MRS: de Man, Rogosa and Sharpe; KAA: Kanamycin Aesculin Azide.

^b^Identified by spot-on-the-lawn method [27] using *Listeria monocytogenes* ATCC 7644 as target.

### Antimicrobial activity and bacteriocin production

From the LAB collection obtained from raw goat milk, 57 isolates presented antimicrobial activity against *L. monocytogenes* ATCC 7644 (Table 2). This foodborne pathogen was selected as a target because previous studies have demonstrated its susceptibility to the antimicrobial substances produced by LAB; it is usually adopted as an indicator of such activity [11,22,25,38,39].

The bacteriocinogenic activity was confirmed by the enzymatic assays in 54 of the 57 antagonistic isolates (Table 2). These isolates produced antimicrobial substances that were degraded by distinct enzymes solutions, mainly by proteinase K and *Streptomyces griseus* protease. The sensitivity to proteases indicated the proteinaceous nature of the produced substances, typical for bacteriocins [13,40]. In addition, the observed results indicated that the bactericions produced by the tested isolates would be degraded by pancreatic enzymes and they would not interfere with the intestinal microbiota of the consumer [41].

It was also observed that some isolates produced antimicrobial substances with sensitivities to α-amylase (7) and lypase (28), suggesting the presence of carbohydrates and lipids in their structures [42,43]. These substances can interfere with bacteriocins stability, demanding further studies to verify their appropriateness as biopreservatives in foods [44].

### Molecular identification and rep-PCR fingerprinting of bacteriocinogenic isolates

All 57 isolates that presented antimicrobial activity against *L. monocytogenes* ATCC 7644, whether they produced antimicrobial substances sensitive to enzymes or not (Table 2), was subjected to molecular identification and rep-PCR fingerprinting. The isolates were identified as *Lactococcus* spp. (24 isolates: 21 *L. lactis* subsp. *lactis*, and 3 *L. lactis*) and *Enterococcus* spp. (33 isolates: 17 *E. durans*, 8 *E. faecalis*, 7 *E. faecium*, and 1 *E. hirae*). For *Lactococcus* spp., it was observed that sequencing of the V1 region (90 bp) of the 16S rRNA gene was sufficient to provide a proper and reliable identification of the isolates, with variations that allowed differentiation of their species and subspecies [29]. However, sequencing of the same region in *Enterococcus* spp. isolates was not enough to provide a reliable identification at the species level, as observed in previous studies [45-48]; this limitation demanded sequencing of the *phe*S gene for a proper identification [30]. Considering the obtained results, isolates from raw goat milk that presented antimicrobial activity were identified as *Lactococcus* spp. and *Enterococcus* spp., as is usually observed in studies that investigate this activity in autochthonous microbiota from food systems [9,11,49].

**Figure 1:**
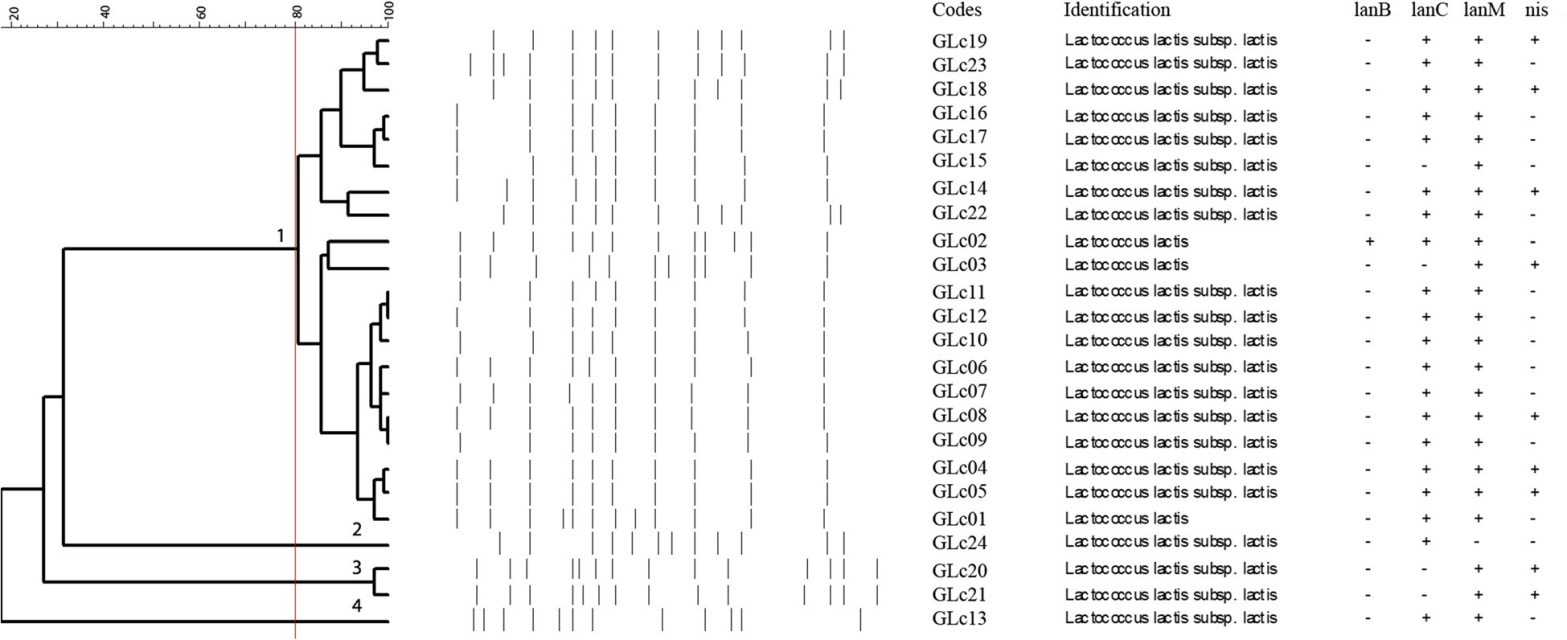
**Dendogram generated after cluster analysis of rep-PCR fingerprints of bacteriocinogenic *Lactococcus *spp. obtained from raw goat milk.** Clusters are indicated by numbers. Presence (+) or absence (-) of bacteriocin encoding genes are also indicated.

**Figure 2:**
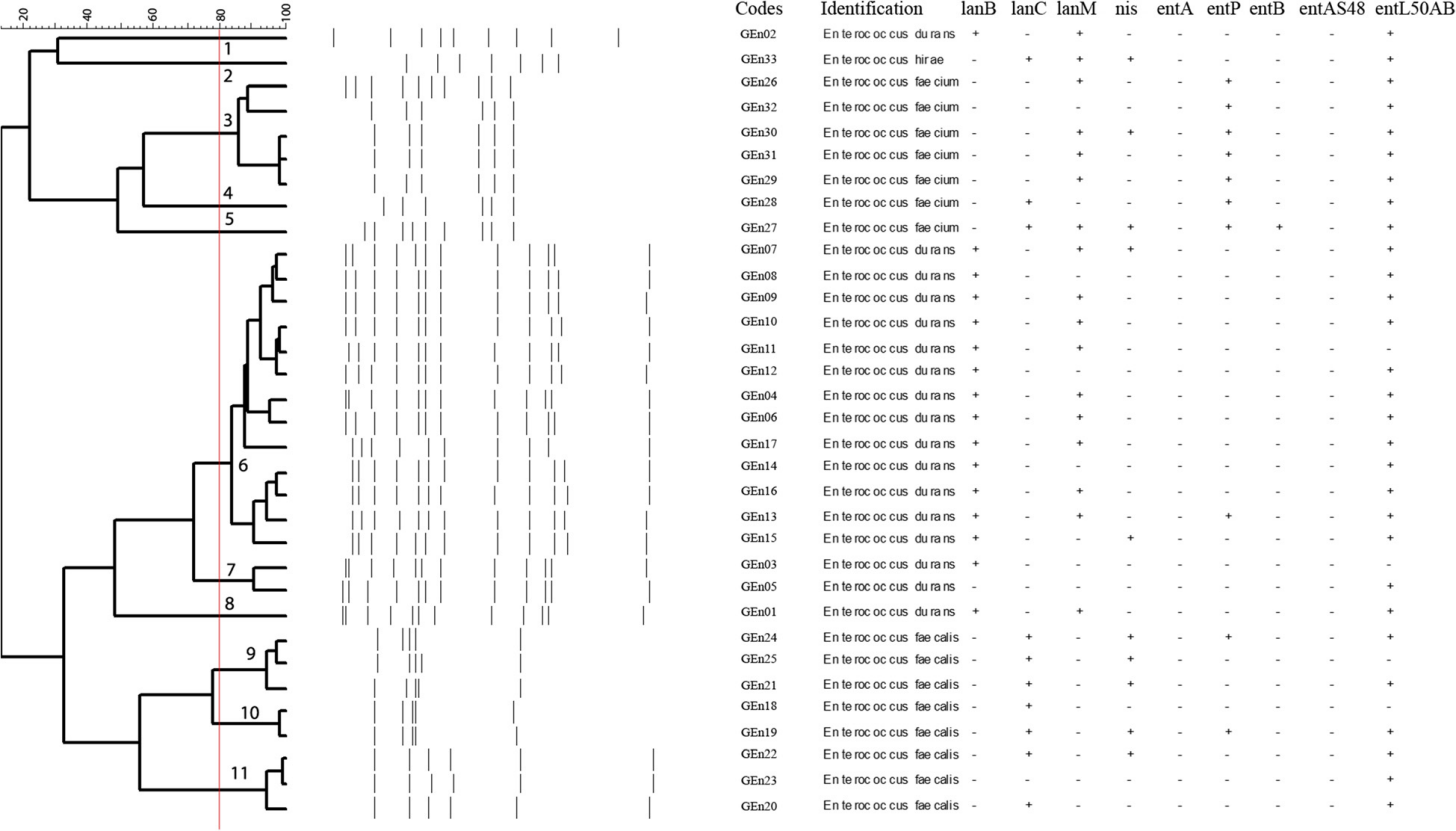
**Dendogram generated after cluster analysis of rep-PCR fingerprints of bacteriocinogenic *Enterococcus *spp. obtained from raw goat milk.** Clusters are indicated by numbers. Presence (+) or absence (-) of bacteriocin encoding genes are also indicated.

For rep-PCR fingerprinting analysis, the isolates were grouped considering their genus identification and 80% similarity to the obtained profiles (Figures 1 and 2). *Lactococcus* spp. isolates were grouped in four clusters, being 20 strains comprising in only one cluster, demonstrating large homology between them (Figure 1). For *Enterococcus*, the isolates were grouped in 11 clusters, demonstrating their biodiversity and evident similarities between isolates from the same species (Figure 2). Rep-PCR has already been described as a reliable methodology to determine the intra-species biodiversity of LAB isolated from foods, and also to assess the genetic variability of bacteriocinogenic strains [9,50,51].

### Bacteriocin encoding genes

Figures 1 and 2 also present the results for bacteriocin encoding genes assessed in the *Lactococcus* spp. and *Enterococcus* spp. isolates, respectively.

All *Lactococcus* spp. isolates presented lantibiotic genes in distinct associations, only one (GLc02) presenting *lan*B, *lan*C and *lan*M simultaneously (Figure 1). *lan*B was the less frequent gene, while *lan*C and *lan*M usually were present simultaneously in the majority of isolates; this result was expected, since both genes are located in the same operon in the bacterial genome [52]. However, the isolated presence of *lan*C or *lan*M has already been described in previous studies [19,25]. For *Enterococcus* isolates, 30 isolates presented at least one of the tested lantibiotic genes; no isolates presented *lan*B, *lan*C and *lan*M simultaneously (Figure 2). Cytolisin is a class I lantibiotic produced by *Enterococcus* spp., a bacteriocin that can be related to the tested genes [53]. Considering the antimicrobial potential of the isolates, the presence of at least one of the tested genes would be sufficient for lantibiotic production [17,19].

A lower frequency of positive results was observed for *nis* in the tested *Lactococcus* isolates (9 strains) compared to similar studies identifying the bacteriocinogenic potential of this genus (Figure 1) [9,22,25,49]. Still considering the results for the *nis* gene, ten *Enterococcus* isolates presented typical PCR amplification products (Figure 2). The occurrence of *Enterococcus* strains possessing nisin-related genes has already been reported, and can be explained by the capability of this genus to acquire new genetic elements [40]. However, positive results for the *nis* gene must not be related to the production of nisin by *Enterococcus* isolates.

No *Enterococcus* isolates presenting encoded genes for enterocin A and enterocin AS-48 (Figure 2). Only a single isolate (GEn27) presented a positive result for the enterocin B gene, and 10 isolates, from five distinct clusters, for the enterocin P gene (Figure 2). Enterocin A and enterocin P are bacteriocins classified in subclass IIa (pediocin-like bacteriocins), with typical high inhibitory activity against *Listeria* spp. [53]. The enterocin L50AB gene was detected in 29 isolates, from all identified genetic profiles (Figure 2); this bacterocin is classified in subclass IIb, characterized by its synthesis without leader peptides and demanding a complex system for transport [54,55].

The three LAB isolates that presented antimicrobial activity but an absence of enzymatic sensitivity in their produced substances (Table 2) were two *Lactococcus* (GLc20 and GLc21) and one *Enterococcus* (GEn27) (Figures 1 and 2). However, the three isolates presented positive results for bacteriocin-related genes, indicating that they were unable to express them. When assessing the bacteriocingeonic potential and activity of LAB, the absence of production of bacteriocins by gene positive strains is a common finding, since bacteriocin production is mediated by a diversity of genetic and environmental factors [13,40].

### Nisin gene sequencing and inhibitory spectrum of nisin positive isolates

**Figure 3:**
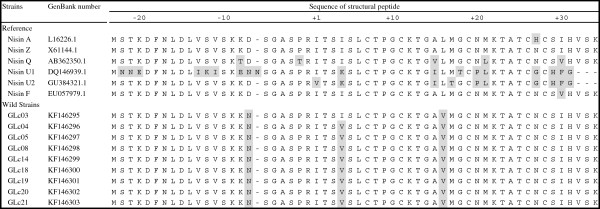
**Amino-acid sequences of a novel nisin variants deduced by the sequencing of nisin region from nine *Lactococcus *spp. strains obtained from raw goat milk and compared to the sequences of nisin A, Z, Q, F and U.** The leader peptide is composed by 23 amino-acids, followed by amino-acids representing the mature peptide. Amino-acids highlighted in grey indicate variations when compared to the nisin A (the first nisin variation to be discovered) references. The complete amino-acid sequencesfrom the 9 wild strains have been deposited in GenBank (accession numbers KF146295 to KF146303, respectively).

The nine *Lactocccus* isolates that presented positive results for *nis* were identified as capable of producing a novel nisin variant. Their amino-acid sequence were diverse from to the other nisin variants already described (Figure 3). In all translated sequences the typical variation in nisin Z was identified: an asparagine instead of a histidine in position 27 (Figure 3), as described previously [25,56]. In addition, all isolates presented identical variations in their translated sequences when compared to a reference sequences of nisin (Figure 3): 1) in the leader peptide, an aspartic acid was replaced by an asparagine in position -7; 2) except for GLc03, an isoleucine was replaced by a valine in position +4; and 3) a leucine was replaced by a valine in position +16 (Figure 3). Concerning the nisin leader peptide sequence, in the position -7, one negative-charged amino-acid (aspartic acid) was replaced by one uncharged amino-acid (asparagine). This same replacement also occurs in Nisin U1 (Figure 3). Indicating that this change cannot interfere with the correct activity of the peptide. It is important to highlight two characteristics: 1) variations in the sequence between positions -18 and -15 would interfere with nisin production, and 2) mutagenesis in Arg^1-^ and Ala^4-^ would affect cleavage of the leader peptide, resulting in a non-active nisin [52]. However, the observed modification in the leader peptide of the translated sequences was not in these regions, indicating that nisin production and activity would not be affected in the tested isolates (Figure 3). Considering the mature peptide, in positions +4 and +16 of the nisin sequence, one neutral amino-acid (isoleucine and leucine respectively) was replaced by other neutral amino-acid (valina). The only described modification in the +4 region is in nisin U (isoleucine replaced by lysine) [19]. The last variation and well know is in position +27, where one uncharged amino-acid (asparagine) is replaced by one positive electrically charge and basic amino-acid (histidin). This typical change for nisin Z was previously described as responsible for increasing its inhibitory spectrum due to its better diffusion capacity in culture media. It is common to observe variations in the amino-acid sequences of lantibiotics, including nisin, that then require proper characterization since they can interfere with the antimicrobial activity of these substances [18]. The observed variations in the translated nisin sequences have not been reported before, after consulting GenBank.

Table 3 shows the inhibitory activity of the *nis* positive *Lactococcus* isolates against several microbial targets. It can be observed that the isolates presented inhibitory activity mainly against the tested Gram positive bacteria, and lower frequencies of inhibition against Gram negative bacteria. These results indicate that the bacteriocins produced by the tested LAB isolates have interesting antimicrobial activities, highlighting the relevance of raw goat milk as a source of bacteriocinogenic strains [23]. In addition, the obtained results indicate that the variations in nisin structure predicted in the present study (Figure 3) did not affect the antimicrobial activity of the isolates. Considering the main characteristics of bacteriocins, the inhibitory activity against the tested Gram negative bacteria must be due to non-specific antimicrobial substances produced by the LAB strains, such as organic acids or peroxide [24,34].

**Table 3:** **Inhibitory activity (diameters of inhibition halos, mm) of *nis *** **positive *Lactococcus *** **isolates obtained from raw goat milk against target microorganisms, identified by the spot-on-the-lawn methodology**

**Target genus**	**Species/serotype**	Origin*	***nis*** **positive isolates**								
			**GLc04**	**GLc05**	**GLc08**	**GLc14**	**GLc18**	**GLc19**	**GLc20**	**GLc21**	**GLc03**
*Lactobacillus*	*L. sakei*	ATCC 15521	11	13	9	9	5	11	0	0	5
*Lactococcus*	*L. lactis* subsp. *lactis*	ATCC 7962	11	9	8	7	0	7	0	0	0
	*L. lactis* subsp. *lactis*	GLc18, wild strain, present study	13	11	11	11	0	12	0	0	7
	*L. lactis* subsp. *lactis*	GLc22, wild strain, present study	13	11	11	7	7	10	7	7	7
*Listeria*	*L. monocytogenes*	ATCC 7644	11	11	11	9	15	13	7	7	9
	*L. monocytogenes*	ATCC 15313	9	9	7	7	0	7	7	5	10
	*L. monocytogenes*	60, wild strain, beef origin	15	14	12	9	7	13	5	5	5
	*L. inoccua*	76, wild strain, beef origin	5	5	5	5	5	5	5	5	9
*Staphylococcus*	*S. aureus*	ATCC 12598	9	7	7	7	7	5	7	7	7
	*S. aureus*	ATCC 14458	9	7	7	7	7	9	11	7	7
	*S. aureus*	ATCC 29213	8	7	7	7	7	7	9	0	7
	*S. aureus*	27AF1, wild strain, cheese origin	9	9	9	7	5	11	7	0	9
	*S. aureus*	27ST1, wild strain, cheese origin	9	9	9	7	5	7	11	7	9
	*S. aureus*	26BP6, wild strain, cheese origin	13	13	14	7	7	13	7	0	7
*Escherichia*	*E. coli*	ATCC 11229	0	0	0	0	0	0	0	0	0
	*E. coli*	ATCC 00171	0	0	0	0	0	0	0	0	0
*Pseudomonas*	*P. aeruginosa*	ATCC 27853	5	5	5	5	0	0	5	0	0
	*P. fluorescens*	ATCC 10038	5	5	5	0	0	0	0	0	0
*Salmonella*	*S.* Typhimurium	ATCC 14028	7	7	5	5	0	0	0	0	0
	*S.* Cholerasuis	38, wild strain, beef origin	0	0	0	0	0	0	0	0	0
	*S.* Enteritidis	258, wild strain, poultry origin	7	7	7	5	5	5	5	5	0
	*S.* Typhi	40, wild strain, beef origin	0	0	0	0	0	0	0	0	0

*ATCC: American Type Culture Collection, Manassas, VA.

## Conclusions

In conclusion, the present study highlighted the diversity of LAB in the raw goat milk microbiota, representing a potential source of novel bacteriocinogenic strains to be further studied concerning their antimicrobial activity. In addition, *Lactococcus* strains were identified as possessing variations in their *nis* gene sequences that would result in production of a nisin variant not yet described, and also possessing a wide inhibitory spectrum.

### Availability of supporting data

The amino-acid and nucleotide sequences for nisin gene from positive *Lactococcus* spp. strains were deposited and available in the GenBank (National Center for Biotechnology Information, http://www.ncbi.nlm.nih.gov/genbank). The accession numbers are KF146295 - KF146303.

## Competing interests

The authors declare that they have no competing interests.

## Authors’ contributions

LMP: AB, MT, ES, FG. LAN: AB, MT, ES, FG. Both authors read and approved the final manuscript.
